# Metformin ameliorates skeletal muscle insulin resistance by inhibiting miR-21 expression in a high-fat dietary rat model

**DOI:** 10.18632/oncotarget.20442

**Published:** 2017-08-24

**Authors:** Jinyang Wang, Yanbin Gao, Lijun Duan, Suhong Wei, Jing Liu, Liming Tian, Jinxing Quan, Qi Zhang, Juxiang Liu, Jinkui Yang

**Affiliations:** ^1^ Department of Endocrinology, Gansu Provincial People's hospital, Lanzhou, China; ^2^ Gansu Provincial Key Laboratory of Endocrine and metabolism, Lanzhou, China; ^3^ School of Traditional Chinese medical, Capital Medical University, Beijing, China; ^4^ Beijing Key Laboratory of TCM Collateral Disease Theory Research, Beijing, China; ^5^ Department of Gynecology and Obstetrics, Gansu Provincial People's Hospital, Lanzhou, China; ^6^ Department of Endocrinology, Beijing Tongren hospital of Capital Medical University, Beijing, China

**Keywords:** miRNA, metformin, insulin resistance (IR), TGF-β/Smads, luciferase reporter gene assay

## Abstract

Insulin resistance (IR) plays a major role in the pathogenesis of abdominal obesity, hypertension, coronary heart disease, atherosclerosis and diabetes. miR-21 and TGF-β/smads is closely related to IR. However, it remained elusive whether metformin improved skeletal muscle insulin resistance (IRSM) by regulating miR-21 and its target signal TGF-β1/smads expression. In this study, high-fat diet rats with IR model and IR-skeletal muscle L6 cells (L6-SMCs) model were established, insulin sensitive index (ISI) and Homeostasis model assessment of IR (HOMA-IR) were applied, miR-21 and TGF-β1/smads mRNA expression were examined by RT-PCR, smad3 and smad7 protein were detected by western-blotting and laser scanning confocal microscopy (LSCM), the valid target of miR-21 was detected by luciferase reporter gene assay. Here, we found that metformin dose-dependently decreased miR-21 expression, accompanied by the decrease of HOMA-IR and the increase of HOMA-ISI. Luciferase report gene assay showed that smad7 was an effective target of miR-21. miR-21 overexpression directly downregulated smad7 and indirectly upregulated smad3 expression. Interestingly, miR-21 expression positively correlated with HOMA-IR and negatively correlated with HOMA-ISI. In conclusion, our results demonstrated that metformin improved IRSM by inhibiting miR-21 expression, and that miR-21 may be one of the therapeutic targets for IR.

## INTRODUCTION

Insulin resistance (IR) is defined as a reduced response of target tissues (skeletal muscle, liver, and adipocytes) to insulin [1]. Skeletal muscle exerts a key role in regulating whole body glucose homeostasis, which is responsible for 70%–80% of insulin-stimulated glucose uptake, skeletal muscle insulin resistance(IRSM) is not only most likely a major determinant of type 2 diabetes, but also plays a major role in the pathogenesis of abdominal obesity, hypertension, coronary heart disease, atherosclerosis and other cardiovascular disease[2]. Emerging evidence suggests that TGF-β/Smads signaling pathway and its downstream signaling molecules (such as smad3 and smad7) plays a pivotal role in the mechanism of IR [3], Smad3 is the promoting factor of IR [4–6], while Smad7 is an inhibitory smad that blocking the function of smad3 [7].

miRNAs, small non-coding RNAs consisting of 20-23 nucleotides, are known to act as powerful post-transcriptional regulators, which act either through the inhibition of protein translation or via mRNA degradation, by partially binding to the 3′UTR of their target mRNAs [8–10]. Recent studies have indicated that miR-21 plays a crucial role in IR and skeletal muscle biological processes, but also associated with TGF-β1/smads signal. With regard to IR and muscle diseases. miR-21 may be as a therapeutic target in burn-induced IR [11]. miR-21 plays an important role in skeletal muscle growth in chicken [12]. miR-21 was involved in bovine skeletal muscle satellite cell myogenic differentiation [13]. miR-21 was involved in the pathogenesis of IR and diabetic mellitus-induced non-alcoholic fatty liver disease [14]. miR-21 reverses high glucose and high insulin induced IR in 3T3-L1 adipocytes through targeting phosphatase and tensin homologue [15]. miR-21 may be a new therapeutic target for metabolic diseases such as T2DM and obesity [15]. As for miR-21 and TGF-β1/smads, smad3-mediated upregulation of miR-21 promotes epithelial to mesenchymal transition (EMT) [16]. MiR-21/smad7 signaling determines TGF-β1-induced CAF formation [17]. TGF-β regulates TGFBIp expression in corneal fibroblasts via miR-21 signaling [18]. More importantly, our previous experiments demonstrated that miR-21 overexpression enhanced TGF-β1-induced renal tubular EMT by inhibiting smad7 [19]. These findings strongly suggested that miR-21 not only participated in IR, but also was closely related to TGF-β1/smads. Due to miRNA's expression had spatio-temporal specificity in different tissues, cells and different phase of diseases, it remained unclear whether miR-21 was involved in IRSM by regulating smad7.

Metformin, a biguanide derivative, is described as an insulin sensitizer, which causes a reduction in IR and a significant decrease in plasma fasting insulin levels [20, 21]. Most excitingly, it was recently demonstrated that metformin not only influences many miRNA expression profile but also has a role in the alteration of miRNA activity in diabetes and cancer field [22–24]. For example, metformin alters the expression profiles of miRNAs in human pancreatic cancer cells [25]. Metformin inhibits epithelial-mesenchymal transition in prostate cancer cells: involvement of the tumor suppressor miR-30a and its target gene SOX4 [26]. Metformin induces growth inhibition and cell cycle arrest by upregulating miR-34a in renal cancer cells [27]. Metformin induces ER stress-dependent apoptosis through miR-708-5p/NNAT pathway in prostate cancer [28]. More importantly, metformin-mediated increase in DICER1 regulates microRNA expression and cellular senescence [29]. These results strongly suggested that metformin was able to modulate miRNA expression and activity to control tumor-related diseases. However, it remained elusive whether metformin improved skeletal muscle insulin resistance (IRSM) by regulating miR-21 and its target signal TGF-β1/smad7 expression.

In the present study, our aim was to determine whether miR-21 was involved in IRSM by regulating smad7 and metformin improved IRSM by inhibiting miR-21 expression. Our results demonstrated that miR-21 was involved in IRSM by directly downregulating smad7 and indirectly upregulating smad3 expression. More importantly, metformin improve IRSM by inhibiting miR-21 expression, and that miR-21 may be one of the therapeutic targets for IR.

## RESULTS

### miR-21 was positively correlated with HOMA-IRI and metformin decreased miR-21 expression in concentration-dependent way

**Figure 1:**
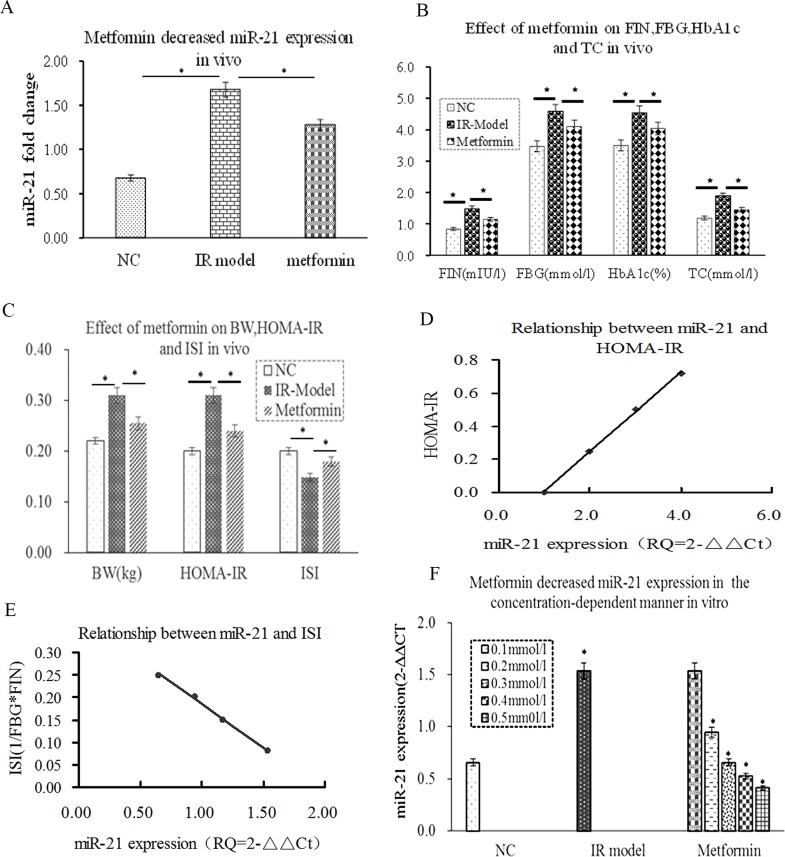
miR-21 was positively correlated with HOMA-IRI and metformin decreased miR-21 expression in concentration-dependent way **(A)** compared with NC group, miR-21 expression was a significant difference in the IR group (P≤0.05). Metformin obviously inhibited miR-21 expression *in vivo* (P≤0.05). **(B)** FIN, FBG, HbA1C and TC were increased in the IR group (P≤0.05). Metformin could decrease the levels of FBG, HbA1C, TC and FIN *in vivo* (P≤0.05). **(C)** the increase of HOMA-IR and BW and the decrease of HOMA-ISI in the IR group (P≤0.05). Metformin could decrease HOMA-IR and BW and increase HOMA-ISI (P≤0.05). **(D)** the level of miR-21 expression was positively correlated with HOMA-IR (r=0.786, p<0.05). **(E)** the level of miR-21 expression was negatively correlated with HOMA-ISI(r=-0.833, p<0.05). **(F)** metformin reduced miR-21 expression in concentration-dependent way (0.2-0.5mmol/l) *in vitro*, whereas, miR-21 expression was unchanged at the concentration of 0.1mmol/l.

HOMA-IR was recognized as a good clinical predictor of IR. HOMA-ISI, which was generally considered as marker of insulin sensitivity index, reflects the insulin sensitive response of target tissues [33]. To confirm the relationship between miR-21 andIR, firstly, miR-21 expression was detected by RT-PCR, the results showed that miR-21 expression was significantly increased in IR model group compared with NC group (Figure 1A, p≤0.01), accompanied by the increase of HOMA-IR, FIN, FBG, HbA1C, BW and TC. In contrast, the decrease of HOMA-ISI (Figure 1B, 1C, p≤0.05). Interestingly, the level of miR-21 expression was positively correlated with HOMA-IR(r=0.786, p≤0.05) and negatively correlated with HOMA-ISI(r=-0.833, p≤0.01) by Pearson analysis (Figure 1D, 1E, p≤0.01). Next, we determined the effect of metformin on miR-21 expression *in vivo* and *in vitro* by RT-PCR, the results showed that metformin could reduce the level of miR-21 expression compared with IR model group, accompanied by the decrease of FIN, HOMA-IR, FBG, HbA1C,BG and TC(Figure 1A, 1B, 1C, p≤0.05). Whereas, the increase of HOMA-ISI (Figure 1C, p≤0.05). More importantly, after L6-SMCs were treated with the different of metformin (0.1-0.5mmol/l), we found that metformin obviously decreased miR-21 expression at the concentration of 0.2-0.5mmol/l, whereas miR-21 expression was unchanged at the concentration of 0.1mmol/l (Figure 1F). Taken together, these results demonstrated that miR-21 expression was closely correlated with IRSM and metformin can ameliorate IR by decreasing miR-21 expression in concentration-dependent way.

### Effect of miR-21 overexpression on TGF-β1/smad3/smad7 expression in L6-SMCs

**Figure 2:**
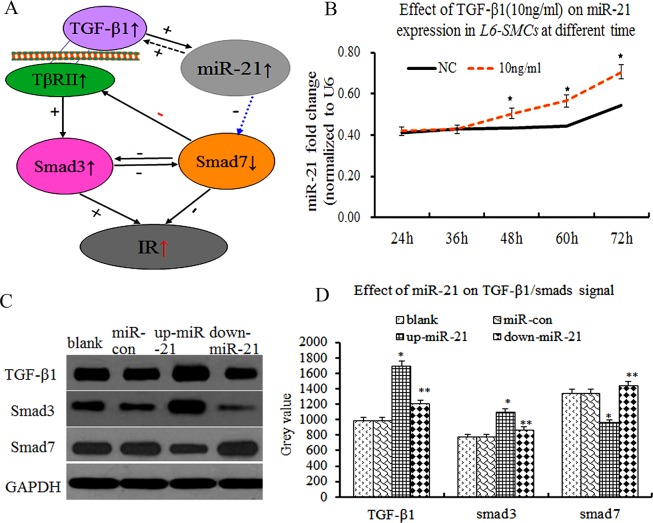
Overexpression of miR-21 enhanced TGF-β1/smad3 and decreased smad7 expression in L6-SMCs **(A)** Scheme showing that the process of miR-21 and TGF-β1 interact to regulate IR, miR-21 is upregulated by TGF-β1, which in turn inhibited Smad7 leading to amplification of TGF-β1 signaling finally resulting in IR. **(B)** the effect of TGF-β1on miR-21 expression in L6-SMCs at different length of time, the results showed that miR-21 expression was significantly elevated in TGF-β1 group (p≤0.05). **(C)** TGF-β1/Smad3 and Smad7 by western blot. **(D)** Comparison of the grey value of TGF-β1/Smad3 and Smad7 protein.

Accumulating evidences have demonstrated that TGF-β1/smads plays an important role in IR, smad3 promoted IR and smad7 inhibited IR in mice [5], suggesting that smad3 and smad7 have an antagonistic effect on IR (Figure 2A). However, the relationship between miR-21 and TGF-β1/smad3/smad7 expression and metformin intervention in L6-SMCs remained unclear. Firstly, to explore the effect of TGF-β1 on miR-21 expression in L6-SMCs, L6-SMCs were treated with TGF-β1 (10ng/ml) at different length of time (24, 36, 48, 60,72h), the results showed that miR-21 expression was significantly elevated in TGF-β1 group compared with NC group at 48h (Figure 2B, p≤0.05). Next, to determine the effect of miR-21 overexpression on TGF-β1/smads *in vitro*, we performed cells transfection experiments, L6-SMCs with the addition of transfection agent and miR-control lentivirus vector (miR-control group), miR-21 overexpression lentivirus vector (pre-miR-21 group or miR-21 overexpression group), miR-21 inhibitor lentivirus vector(down-miR-21 group) and L6-SMCs without transfection were used as blank control group (blank group). After 72h transfection, TGF-β1/smad3 and smad7 expression were examined by western blotting. There were the increase of TGF-β1/smad3 and the decrease of smad7 expression in miR-21 overexpression group compared with miR-control group and blank control group (Figure 2C, 2D, p≤0.05). Inversely, there were the decrease of TGF-β1/smad3 and the increase of smad7 expression in down-miR-21 group (Figure 2C, 2D, p<0.05), suggesting that miR-21 over-expression could downregulate smad7 and upregulate smad3 expression, and that miR-21 can result in the degradation of smad7 and further lead to amplification of TGF-β1/Smad3 signaling. Thus, we speculated that miR-21 and TGF-β1/Smad3 formed a double-positive feedback loop to enhance IR by downregulating smad7 expression,

### Smad7, but not TGF-β1/ Smad3, was a validated miR-21 target in skeletal muscle cells

**Figure 3:**
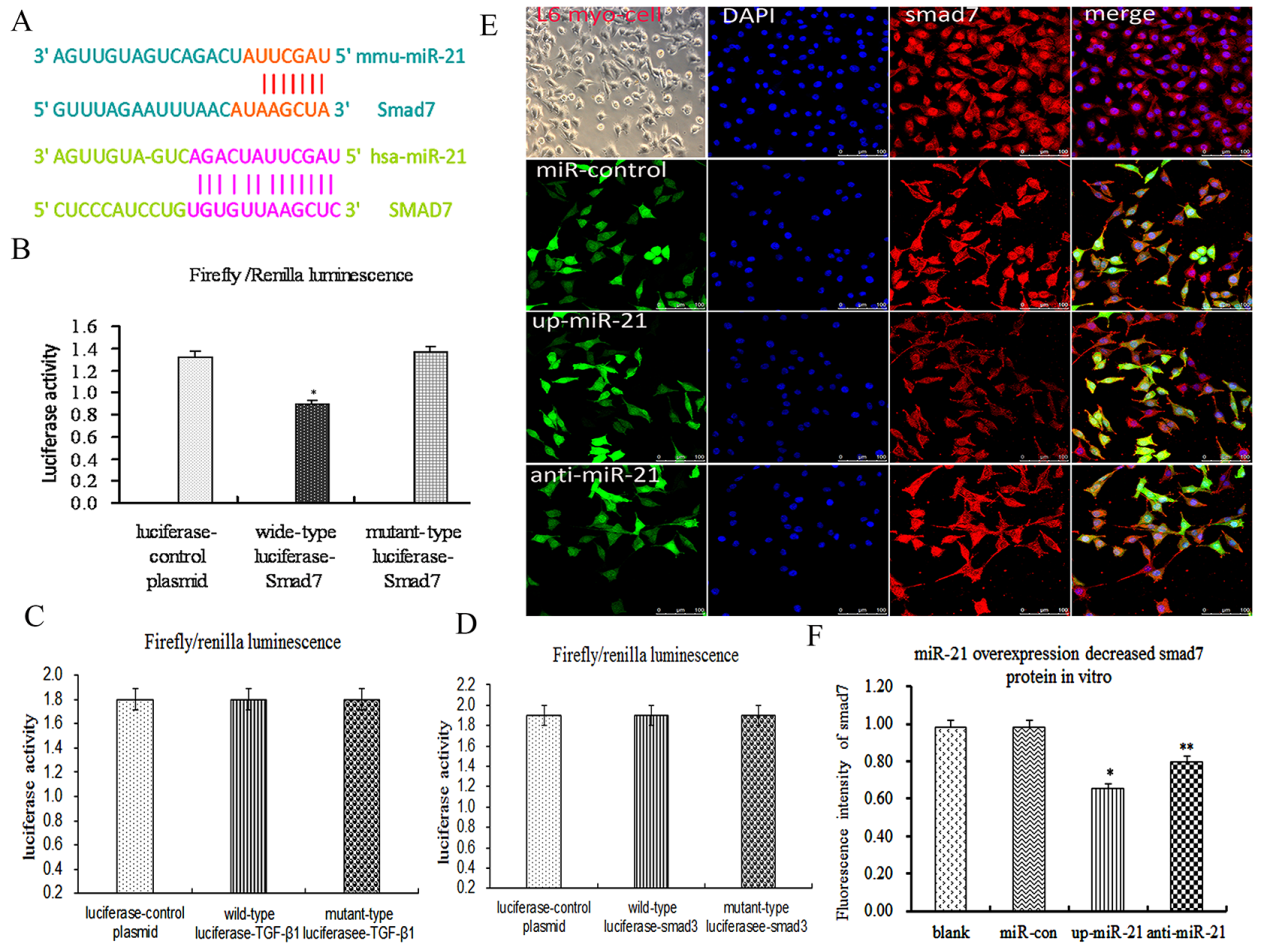
Smad7, but not TGF-β1/Smad3, was a validated miR-21 target in skeletal muscle cells **(A)** Alignment of hsa-miR-21 and mmu-miR-21 with human smad7 3′-UTR and mouse smad7 3′-UTR based on targetScan software, several nucleotides in the 5′-region of miR-21 (human and mouse) contain a perfect match with the 3′-UTR sequence of the human and mouse smad7 genes. **(B)** The results of luciferase report gene assays of smad7 (p<0.05). **(C)** The results of luciferase report gene assays of TGF-β1. **(D)** The results of luciferase report gene assays of smad3 **(E)** Representative photograph of smad7 protein by ICC. **(F)** The fluorescence intensity of smad7 proteins (p<0.05).

As described above, miR-21 over-expression decreased smad7 expression *in vitro*, then, miR-21 over-expression was how to decrease smad7 expression. According to TargetScan database (http://www.targetscan.org/), Smad7, but not TGF-β_1_and Smad3, was a potential target of miR-21(Figure 3A). Therefore, to further confirm whether smad7 was a validated miR-21 target in L6-SMCs, we performed the luciferase report gene assays. The results exhibited that wild-type luciferase-smad7-3′UTR reporter gene for luciferase activity was remarkably decreased compared with mutant luciferase-smad7-3′UTR reporter and control plasmid, suggested that smad7 was a validated miR-21 target in L6-SMCs (Figure 3B, p<0.05). Meantime, to further confirm whether TGF-β1/smad3 was a validated miR-21 target in L6-SMCs, we performed the luciferase report gene assays for TGF-β1/smad3. The results exhibited that wild-type luciferase-TGF-β1/smad3-3′UTR reporter gene for luciferase activity was no difference compared with mutant luciferase-TGF-β1/smad3-3′UTR reporter and control plasmid, suggested that TGF-β1/smad3 was not a validated miR-21 target in MFCs (Figure 3C, 3D, p>0.05). Next, to further testify the function of miR-21, smad7 expression was examined by fluorescent immunohistochemistry (FIHC), the results showed that miR-21 overexpression inhibited smad7 and anti-miR-21 upregulated smad7 protein expression (Figure 3E, 3F, p<0.05). Overall, our results demonstrated that smad7 was a validated target of miR-21, which could directly down-regulate smad7 expression.

### Metformin ameliorated insulin resistance by upregulating smad7 expression of miR-21 target

**Figure 4:**
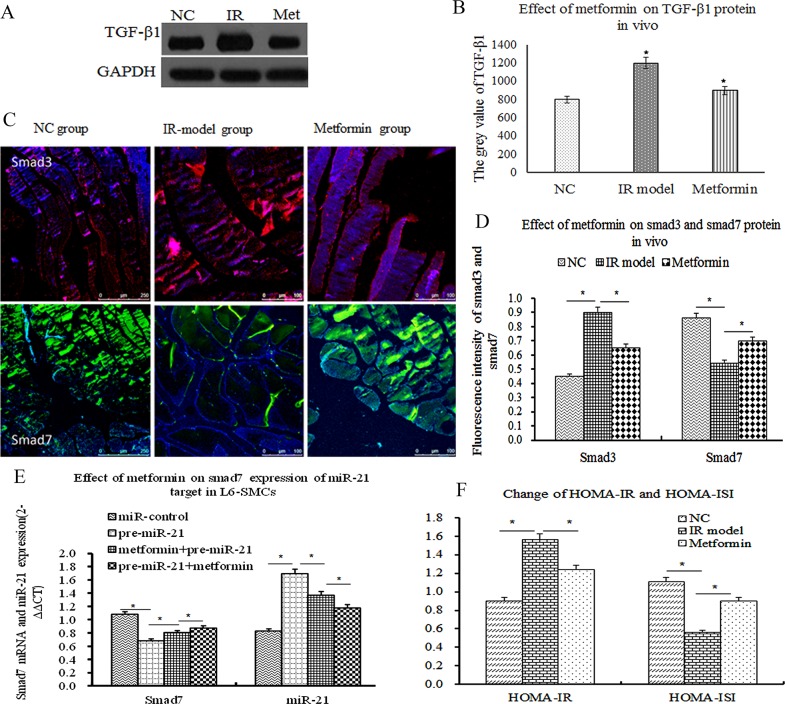
Metformin ameliorated insulin resistance by upregulating smad7 expression of miR-21 target **(A)** TGF-β1 expression by western blot *in vivo*. **(B)** Comparison of the grey value of TGF-β1 protein. **(C)** Representative Photographs of smad3 and smad7 protein by ICC, positive smad3 expression was mainly located in skeletal muscle cells **(D)** The fluorescence intensity of smad3 and smad7 proteins (p<0.05). **(E)** the effect of metformin on smad7 expression of miR-21 target in L6-SMCs, before and after the treatment of metformin, transfected with miR-control and miR-21 over-expression lentivirus vector, the results demonstrated that metformin can remarkably inhibit the decrease of miR-21 overexpression induced-smad7 mRNA. **(F)** The change of HOMA-IR and HOMA-ISI (P<0.05).

As mentioned above, smad3 positively activates and smad7 negatively inhibits signal transduction which mediates IR of skeletal muscle [34]. To determine whether metformin has effect on TGF-β1/smad3 and smad7 expression by inhibiting miR-21 expression *in vivo*, the expression of TGF-β1/smad3 and smad7 were measured by WB and/or ICC at the end of the study. The results showed that TGF-β1/smad3 was significantly increased in the IR model group, In contrast, the expression of smad7 were significantly decreased (Figure 4A, 4B, 4C, 4D, P<0.05). After the treatment of metformin (100mg/kg.day, garvage) for 4 weeks, TGF-β1/smad3 expression were obviously decreased. Conversely, smad7 expression were significantly increased (Figure 4A, 4B, 4C 4D, P<0.05), accompanied by the decrease of HOMA-IR and the increase of HOMA-ISI (Figure 4E, P<0.05). Next, to further investigate the effect of metformin on smad7 expression of miR-21 target in L6-SMCs, before the treatment of metformin(0.2mmol/l), L6-SMCs were transfected with miR-control and miR-21 over-expression lentivirus vector, compared with miR-control group, miR-21 over-expression significantly decreased smad7 mRNA *in vitro* (Figure 4D, P<0.05). After the treatment of metformin(0.2mmol/l) for 48h, metformin-treated L6-SMCs were transfected with miR-21 over-expression lentivirus vector, the results demonstrated that metformin could remarkably inhibit the decrease of miR-21 overexpression induced-smad7 mRNA. Taken together, these results suggested that metformin effectively ameliorated IR by directly upregulating smad7 expression of miR-21 target and indirectly downregulated TGF-β1/smad3 expression.

## DISCUSSION

Insulin resistance (IR) constitutes a common and broadly prevalent metabolic disorder, which seems to govern the pathophysiology of diabetes mellitus, metabolic syndrome, and obesity [1]. Furthermore, IR appears to be a clinically important manifestation of various endocrine diseases, including polycystic ovary syndrome (PCOS), thyroid and adrenal diseases, as well as their complications [35]. From a pathophysiological point of view, IR appears to be the end result of a complex interaction between genetic predisposition and environmental factors [36]. Despite the considerable body of evidence supporting that genetic predisposition and environmental factors are involved in the pathogenesis of IR, the exact underlying mechanisms have not been fully delineated. Previous studies have indicated that many miRNAs play a critical role in regulating IR [11, 37]. Furthermore, computational methods for predicting potential disease-miRNA associations have gained a lot of attention based on their feasibility, guidance and effectiveness [38–40]. Recent studies have indicated that miR-21 plays a crucial role in IR and skeletal muscle biological processes. However, it remained elusive whether miR-21 was involved in IRSM. Our RT-PCR results exhibited that miR-21 expression was significantly increased in IR group, accompanied by the increase of HOMA-IR and the decrease of HOMA-ISI. Interestingly, the level of miR-21 expression was positively correlated with HOMA-IR and negatively correlated with HOMA-ISI. Thereby, we speculated that miR-21 expression was closely correlated with IRSM.

Accumulating evidences demonstrated that TGF-β1/smads signal and miR-21 existed in a complex regulation relationship, and play a pivotal role in IR [5, 19], smad3 and smad7 have an antagonistic effect on IR [16]. However, miR-21 was how to affect TGF-β1/smad3 and smad7 expression in L6-SMCs remained unclear. To determine the between miR-21 with TGF-β1/smads signal in L6-SMCs, our experiment results showed that TGF-β1 could increase miR-21 expression, and miR-21 overexpression could increase TGF-β1/smad3 and decrease smad7 expression, consistent with the previous experiment [41]. More importantly, luciferase report gene assays showed that smad7 was a validated miR-21 target in L6-SMCs. Taken together, our data demonstrated that miR-21 over-expression could directly down-regulate smad7 and indirectly up-regulate smad3 expression, and that miR-21 can result in the degradation of smad7 and further lead to amplification of TGF-β1/Smad3 signaling. Thus, we concluded that miR-21 and TGF-β1/smad3 formed a double-positive feedback loop to enhance IRSM by inhibiting smad7.

Recent evidences have shown that metformin not only influences many miRNA expression profile but also has a role in the alteration of miRNA activity in diabetes and cancer [22]. In our experiment, firstly, to determine the effect of metformin on miR-21 expression *in vivo* and *in vitro*, the results showed that metformin can reduce the level of miR-21 expression. More importantly, metformin (at the concentration of 0.2-0.5mmol/l) can decrease miR-21 expression in concentration-dependent way. Next, to further investigate the effect of metformin on smad7 expression of miR-21 target in L6-SMCs, before and after the treatment of metformin, L6-SMCs were transfected with miR-control and miR-21 over-expression lentivirus vector, compared with miR-control group, miR-21 over-expression significantly decreased smad7 mRNA *in vitro*. The results demonstrated that metformin can remarkably inhibit the decrease of miR-21 overexpression induced-smad7 mRNA. Taken together, these results suggested that metformin effectively ameliorated IR by directly upregulating smad7 expression of miR-21 target and indirectly downregulating smad3 expression, and that miR-21 may be one of therapeutic targets of metformin for amelioratings IR.

In summary, our data demonstrated that miR-21 was involved in IRSM by directly downregulating smad7 and indirectly up-regulating smad3 expression. More importantly, metformin ameliorated IRSM by inhibiting miR-21 expression, and inhibition of miR-21 may be an effective target for directly alleviating IR.

## MATERIALS AND METHODS

### Cell culture and induction of insulin resistance

Rat skeletal muscle (L6) cells (L6-SMCs) were obtained from Chinese Type Culture Collection (CTCC). cells were maintained in MEM (Sigma, USA) supplemented with 10% fetal bovine serum (FBS), 100U/ml penicillin, and 100μg/ml streptomycin at 37°C in a humidified atmosphere with 5%CO2. L6 cells were used for experiments after 14 days of differentiation, and differentiation was monitored by the appearance of closely aligned and fused myotubes. To induce IR in muscle cells, we treated differentiated L6-SMCs with 750μmol/L palmitic acid for 14 hours according to a method reported by Sawada et al [30]. L6-SMCs were divided into three groups as followed: cells were maintained in MEM as normal control group (NC group), cells were treated with palmitic acid as IR model group (IR model group), and cells were treated with palmitic acid plus 0.2mmol/L metformin as metformin treatment group (metformin group, Met group). Meantime, miR-21 expression was detected at the different concentration of metformin (0.1-0.5mmol/l). In addition, effect of TGF-β1 (10ng/ml) on miR-21 expression was detected in L6-SMCs at the time of time (24, 36, 48, 60,72h). Cells was treated with 10 ng/ml TGF-β1 as TGF-β1 group.

### Animal model and experimental design

30 Male Sprague-Dawley rats, aged 10 weeks and weighed 180 to 250g were purchased from Chinese Academy of Medical Sciences (Beijing, China), The animals were housed in a controlled environment (24±1°C, 12h light: 12h dark cycle) an protocol allowed to food and water adlibitum. We followed standard animal experimental procedures approved by the Animal Ethics Committee. After 3-day acclimatization, 30 of the whole were randomly divided into two groups: Normal control group (NC group, n=10) and high-fat diet group (HF group, n=20). To induce IR model, NC group were fed by common forage (12% fat, 60% carbohydrate, and 28% protein), HF group were received research diets (58% fat, 25.6% carbohydrate, and 16.4% protein) for 20 weeks, FBG, Fasting insulin (FINS,mIU/L), HbA1c, total cholesterol (T-Cho,mmol/l) and Body weight (BW) were detected. HOMA insulin resistance index (HOMA-IR) was calculated as [FBG (mmol/L)^*^FINS (mIU/L)]/22.5. HOMA insulin sensitivity index (HOMA-ISI) was calculated as 1/ (FBG^*^FINS). When HOMA-IR and HOMA-ISI were significantly differences between HF group and NC group (P≤0.05), HF rats (20 male rats) were randomly divided into two groups: IR model group (n=10), and metformin group (100mg/kg.day, garvage, n=10) for 4 weeks. Animals were sacrificed at 34 weeks, skeletal muscle tissue from each rat for western blot, RT-PCR and immulohistochemical staining, respectively.

### Real-time RT-PCR analysis

Total RNA from tissue and cells were isolated using TRIzol reagent (Invitrogen) to obtain both miRNA and mRNA. Real-time PCR primers were designed as described previously [31]. Relative expression was calculated using the 2-ΔΔCT method [32] and normalized to the expression of U6 RNA. The relative expression for TGF-ß1, smad3, smad7 was normalized to the expression of ß-actin. Primers for real-time PCR: miR-21: Forward primer (F): 5′-gggtagcttatcagactgatgtt-3′, Reverse primer(R):5′-cagtgcagggtccgaggt-3′;TGF-β1:F:5′- tgccctctacaaccaacacaacccg-3′, R: 5′-aactgctccaccttgggcttgcgac-3′; smad3: F: 5′-agggctttgaggctgtctacc-3′, R: 5′-gtccacgctggcatcttctg-3′; smad7: F: 5′-ttttgaggtgtggtggg-3′, R:5′-gaggcagtaagacagggatga-3′; All Real-time RT-PCRs were performed at least 3 separate times in triplicate and the data are presented as mean±SD.

### Western blot analysis

Protein samples were subjected to 10% SDS-polyacrylamide gel electrophoresis and then transferred to PVDF membranes. The PVDF membranes were blocked with 50% skimmed milk, treated with primary antibody at 4°C overnight, washed and then incubated with the secondary horseradish for two hours. Bands were detected with Enhanced Chemiluminescence (ECL). Immunoblotting was performed with rabbit polyclonal to TGF-β1 antibody (Abcam, 1:200), rabbit monoclonal to smad3 (1:500; Abcam), rabbit monoclonal to rabbit monoclonal to smad7 antibody (1:500, Epitomics). Then, membranes were incubated with the secondary horseradish (1:5000) and exposed to X-ray. Densitometry was detected by Imagine J. Western blot analyses were performed at least in triplicate.

### Immunocytochemistry (ICC) and immunohistochemistry

Cells were incubated on the coverglasses in six orifice plates, and then fixed with 4% paraformaldehyde. Antibodies and dilutions were as follows: rabbit monoclonal to smad3 (1:500; Abcam) and rabbit monoclonal to smad7 antibody (1:500, Epitomics). The cells were then incubated with the secondary antibody for two hours. DAPI was used to stain the cell nuclei (blue). Cells were observed under the confocal microscope (Leica TCS SP5 MP, Heidelberg GmbH,German). For *in vivo* studies, kidney tissue sections (4μm) were subjected to immunohistochemical staining (IHC) for smad3 and smad7. The percentage of positively-stained area with 40 fields of view was analyzed by Image-pro plus 6.0 (Media cybernetics).

### Luciferase reporter gene assays

To examine whether smad7 was a validated target of miR-21, a putative single copy of miR-21-recognition element from the 3′-UTR of smad7 gene was cloned into downstream of the dual luciferase reporter gene of GV306 plasmid vector (Genechem, Shanghai, China), L6-SMCs were co-transfected with the GV306 vector containing smad7 3′-UTR and miR-21 overexpression plasmid by Lipofectamine 2000 (Invitrogen, Carlsbad, CA, USA), and the co-transfection with non-targeting negative control RNA was performed as control. At 48h post transfection, cells were lysed and assayed for luciferase activity with a dual luciferase reporter assay kit (Promega, Madison, WI, USA) on a luminometer (Lumat LB9507). Additionally, TGF-β1/smad3-3′UTR reporter gene for luciferase activity was detected, to further identify whether TGF-β1/smad3 was a validated target of miR-21.

### Cells transfection experiments

For transfection experiments, L6-SMCs were seeded at a density of 2×10^4^ cells/cm^2^ in serum-free MEM, with the addition of transfection agent and miR-control lentivirus vector (miR-control group), miR-21 overexpression lentivirus vector (pre-miR-21 group or miR-21 overexpression group), miR-21 inhibitor lentivirus vector(down-miR-21 group) and L6-SMCs without transfection were used as blank group (blank control group). After 12h transfection, medium was changed and L6-SMCs were incubated with fresh serum-containing medium for another 48-72h. Additionally, we observed that metformin whether affects the expression of miR-21. Before the transfection, L6-SMCs were treated with 0.2mmol/l metformin for 72h, and then, pre-miR-21 lentivirus vector was transfected into L6-SMCs as metformin+pre-miR-21 group. After the transfection of pre-miR-21 lentivirus vector, cells treated with 0.2mmol/l metformin for 72h as pre-miR-21+metformin group. All transfections were performed with the aid of ploybrane transfection agent (Genechem, Shanghai, China), following the manufacturer's instructions. The entire abovementioned lentivirus vector was custom-synthesized by Shanghai Genechem Co., Ltd, China.

### Statistical analysis

Statistical analysis was performed using SPSS 16.0 software (IBM, USA). Values are expressed as mean±SD. Differences between groups were calculated using analysis of variance (ANOVA). Differences between two groups were calculated using the Tuckey-test. P≤0.05 was defined as significant.

## Abbreviations

FBG: Fasting blood glucose
FINS: Fasting insulin
T-Cho: total cholesterol
HbA1c: glycosylated hemoglobinA1c
IRSM: skeletal muscle insulin resistance
BW: Body weight
HOMA-IR: Homeostasis model assessment of insulin resistance
HOMA-IR: HOMA insulin resistance index was calculated as [Plasma glucose (GLU, mmol/L)×serum insulin (mIU/L)]/22.5
HOMA-ISI: HOMA insulin sensitivity index was calculated as 1/(GLU×serum insulin)
